# Selections

**Published:** 1881-12

**Authors:** 


### Selections.

### THE CASE OF PRESIDENT GARFIELD. By FRANK H. HAMILTON, A.M. M.D., LL.D.

Relative positions of the President and the Assassin—Point of entrance of Ball—Course of the Ball—Location of the Ball—Symptoms immediately following the Shooting—Supposed Course of the Ball—Symptoms pointing to right Iliac Fossa as the location of the Ball—Formation of Pouch of Pus in, and Ossification of Main Channel—Incision below twelfth Rib—Irritability of Stomach—Parotitis—Bronchitis and Broncho-Pneumonia—Atmospheric Influences—Removal to Long Branch—Death—Official Report of Autopsy—Additional Facts in relation to Autopsy—The Impracticability of determining the Course of and extracting the Ball—General Principles established in Gun-Shot Wounds of Belly—Application of Lister's Dressings to the President's Wounds—Impropriety of making a Counter-opening in the long Sinus.

In answer to our inquiries, Dr. Hamilton dictated as follows:

So far as I am informed, the testimony is conflicting as to the relative positions of the President and the assassin when the pistol was fired. It is now rendered probable that the assassin stood well to the right and slightly in the rear of the President. The ball entered about four inches to the right of the spine, penetrating and comminuting the eleventh rib, entering the intervertebral substance between the last dorsal and first lumbar vertebræ, and passing obliquely forward, emerged at a point near the center of the first lumbar vertebra in front; and was found some distance to the left of the vertebra at the lower margin of the pancreas—being situated nearer its posterior than its anterior surface—wholly without the peritoneal cavity. It is unneeessary to say that the course of the ball, after penetrating the rib, was not determined until after death. I saw the patient on the morning of July 4, in consultation. We were then informed of the manner of the accident, and that on the receipt of the injury the President had fallen to the floor, sinking down to the right side; that, being interrogated, he complained of pain in his right ankle, and subsequently, in the course of the day, of a similar pain in his left ankle; which pains had been promptly relieved by the hypodermic injection of morphine. He vomited immediately after the receipt of the injury, and in the course of the day his urine had to be drawn once by the catheter. There was not, when first seen by myself, nor has there been at any time subsequently, any apparent loss of power in his lower extremities, or diminution of the natural sensibility at any point. The pains in his ankles, however, were accompanied with hyperæsthæsia of the integument; and a few days later it was observed that there was hyperæsthesia of the integument of the right side of the scrotum. All of these symptoms—the pain and the hyperæsthesia—disappeared wholly in the course of the first week or two, and never returned. On the morning of the fourth of July, the patient, being partially under the influence of the morphine, was not suffering pain, the bowels were tympanitic, and the pulse was feeble. At the first consultation, the question having arisen as to the probable course of the ball, it was stated that Surgeon-General Wales, of the Navy, had on the day of the receipt of the injury introduced his finger to its full extent, and that he had declared that it penetrated the substance of the liver, the structure of which he recognized by its granular feel; and Dr. Bliss stated that he had introduced a probe about three inches, which seemed to have passed in the same direction. This testimony was regarded sufficient to determine that the ball was at least beyond our reach, and beyond the reach of safe exploration. Dr. Woodward had introduced his finger sufficiently deep into the wound to determine that the rib was broken. Finding upon personal examination and inspection that the track of the wound was completely closed by a firm clot, I refused to make any further exploration. From this time forward great uncertainty existed in the minds of the medical attendants as to the actual course and present situation of the ball. On the 24th of July, and after the complete subsidence of the tympanites, a circumscribed point of induration was discovered in the right iliac fossa, which at once led to a suspicion that the ball had been deflected, coursing along the anterior surface of the lumbar muscles, and that this induration indicated its present seat. This suspicion was sustained by the hyperæsthesia of the right side of the scrotum, which, as Prof. Weisse had already shown in his anatomical observations, would be the natural result of an injury of the ileo-inguinal or ileo-hypogastric nerves, which lie in the course of the then supposed track of the ball. Still further confirmation was added when, on the 27th of July, we found that a flexible catheter could be carried downward in the direction of the supposed situation of the ball to a distance of seven inches. The point of induration in the right iliac fossa gradually moved downward and became more hard and defined, conveying the impression that it was the ball and that it was encysted. At the autopsy, it having been determined that this was not the ball, further examination of the channel in this direction was not prosecuted. Indeed, this induration had entirely disappeared after death, and it is now presumed that it only indicated the lower end of the long sinus already described.

About this period a small pouch of pus was formed in connection with the main channel, extending underneath the integuments of the back, causing rigors, which were at once relieved by a free incision ; and a little later rigors followed in consequence of the temporary obstruction of the channel caused by the floating of a small fragment of the rib into the orifice, which was relieved on the removal of the fragment.

On the 8th of August, great difficulty having been experienced in the introduction of the drainage tube into this long suppurating canal, an incision was made below the twelfth rib, the patient being under the influence of ether. About a week later, the stomach of the President became exceedingly irritable, and it was found necessary to suspend alimentation by the mouth, and for three or four days he was nourished only by enemata. On the fourth day after the suspension of alimentation by the mouth the right parotid gland began to enlarge (Aug. 17), and on Aug. 24 suppurated and was incised ; the first incision giving exit only to a few drops of pus. Subsequently it opened into the mouth and meatus auditorius externus, and three or four incisions were made at different points on the surface for the exit of matter. At the time of death the suppuration and swelling of the parotid gland had almost entirely disappeared.

Following the parotitis there was a gradual development of bronchitis in the right lung ; and finally a broncho pneumonia of the lower portion of the right lung, indicated by well defined dullness and a total absence of the respiratory murmur in that region. From this time until the period of his removal from Washington there are no events of striking interest worthy of being related in this brief summary, except the alarming weakness and great somnolency of the patient, which occurred on the 24th, 25th and 26th of August, and which led to an apprehension that a fatal issue was at hand. The patient was evidently suffering from atmospheric influences, the heat being intense and oppressive, and most of the time the air being motionless, so that not a leaf could be seen to stir upon the trees surrounding the White House. There was no evidence, however, at any time, that the patient suffered from malaria having its source in the house drainage or the marshes in the vicinity, and which latter at a later time in the season had always been regarded as pestiferous. His removal to Long Branch occurred on the 6th of September, and was effected without injury or discomfort to the patient, with only a slight amount of fatigue, manifested after his arrival, and from which on the following morning he had completely recovered. There was no day while he lay in the cottage at Long Branch that he did not express himself as pleased and even delighted with the change; nor was he ever oppressed by the heat, although one of the days, the first after his arrival, was the hottest day of the season. At two o'clock in the afternoon of this day, when the heat was greatest, in reply to an inquiry, he said he experienced no discomfort. From this time until the period of his death, which was sudden and unexpected, although in no sense unanticipated, there is no incident worthy of special note—except that there was a gradual change in the last two or three days for the worse. The manner of his death and the result of the subsequent autopsy are sufficiently explained in the official bulletin.

[We here insert the official bulletin.— ED.

"A post-mortem examination of the body of President Garfield was made eighteen hours after death, in the presence and with the assistance of Drs. Hamilton, Agnew, Bliss, Barnes, Woodward, Reyburn, Andrew H. Smith, of Elberon, and Acting Assistant Surgeon D. S. Lamb, of the Army Medical Museum, Washington. The operation was performed by Dr. Lamb. It was found that the ball, after fracturing the right eleventh rib, had passed through the spinal column in front of the spinal canal, fracturing the body of the first lumbar vertebra, driving a number of small fragments of bone into the adjacent soft parts, and lodging below the pancreas, about two inches and a half to the left of the spine and behind the peritoneum, where it had become completely encysted. The immediate cause of death was secondary hæmorrhage from one of the mesenteric arteries adjoining the track of the ball, the blood rupturing the peritoneum, and nearly a pint escaping into the abdominal cavity. This hæmorrhage is believed to have been the cause of the severe pain in the lower part of the chest complained of just before death. An abscess cavity, six inches by four in dimension, was found in the vicinity of the gall bladder, between the liver and the transverse colon, which were strongly adherent. It did not involve the substance of the liver, and no communication was found between it and the wound. A long suppurating channel extended from the external wound, between the loin muscles and the right kidney, almost to the right groin. This channel, now known to be due to the burrowing of pus from the wound, was supposed during life to have been the track of the ball. On an examination of the organs of the chest evidences of severe bronchitis were found on both sides, with broncho-pneumonia of the lower portions of the right lung, and, though to a much less extent, of the left. The lungs contained no abscesses, and the heart no clots. The liver was enlarged and fatty, but free from abscesses. Nor were any found in any other organ except the left kidney, which contained near its surface a small abscess, about one-third of an inch in diameter. In reviewing the history of the case in connection with the autopsy, it is quite evident that the different suppurating surfaces, and especially the fractured, spongy tissue of the vertebra, furnish a sufficient explanation of the septic condition which existed.

D. W. BLISS,
J. K. BARNES,
J. J. WOODWARD,
ROBERT REYBURN,
FRANK H. HAMILTON,
D. HAYES AGNEW,
ANDREW H. SMITH,
D. B. LAMB."]

It may be necessary, however, to repeat, inasmuch as contrary statements have been made, that the lungs contained not even the most minute abscess, and that there was no metastatic abscess found in any of the structures examined, except one less than a half inch in diameter near the surface of the left kidney. There were three small serous cysts under the peritoneal covering on the convex edge of the right kidney, each about the size of the vertical section of a large pea. The abscess found between the transverse colon and the liver was evidently not metastatic, but probably was caused by the original injury. There was no cicatrix or wound of the liver, nor anything to indicate that it had suffered injury in the slightest degree.

Since it has been thought by some that it was the duty of the surgeons to have ascertained positively the course and location of the ball, it is proper to consider whether either the one or the other were practicable.

As to determining the course of the ball by a probe, every anatomist will see that it was impossible—if he will consider the very tortuous course which the ball must have taken to reach its final destination; that it passed through the solid structure of the vertebra, and that no metallic instrument sufficiently firm to give indications of the course and direction which it took within the body could ever have reached the ball; nor would any surgeon of experience, familiar with gun-shot wounds in the belly, in the absence of any satisfactory or conclusive evidence as to what course the ball had taken, venture to introduce a probe into the abdominal cavity for the purpose of exploring the supposed rack; nor, indeed, if he had evidence as to the course and situation of the ball, could he have been justified in such an exploration. No point is better settled in surgery than that interference of this sort in gun-shot wounds of the belly is meddlesome, useless and dangerous; and had it been done, and a fatal peritonitis in consequence been set up, the surgeon doing it would have been justly held responsible for the fatal result.

As to the possibility of the extraction of the ball safely, it would have required a large tegumentary and muscular incision as a means of approach to the spinal column; the actual removal of the whole of the twelfth lumbar vertebra in order to furnish a sufficient channel through which the bold surgeon should advance with his instrument for extraction; and after emerging from the cavity thus made in the spinal column, he would have to penetrate or grope his way cautiously between the ganglionic system of nerves, and arteries, veins, lymphatics, including the thoracic duct, all of which are vital structures almost inextricably joined to each other on the front and sides of the spinal column, and the lesion of any one of which must have proved inevitably fatal.

Throughout the whole course of the treatment, contrary to what has been publicly said repeatedly, so far as it was possible to apply the system of antiseptic surgery advocated by Mr. Lister to a wound of this character, it was rigorously employed.

I am reminded now to say, in reply to some suggestions made from time to time, that we ought to have made a counter-opening in the lower portion of the long sinus which terminated in the right iliac fossa; that there was no period of time during the progress of the case in which we felt absolutely certain that what we recognized in the fossa as a point of induration was the ball; nor were we entirely certain at any time where the lower end of the sinus was actually situated; nothing but a very flexible instrument could ever be introduced, and inasmuch as when introduced, its presence in the track could not be recognized by the sense of touch, we were left without any means of determining, with a sufficient degree of accuracy to justify an operation, where the lower end of the channel was. Indeed, it is probable that the flexible catheter employed never reached the lower end of the channel, but doubled upon itself near the crest of the ileum. To have cut through, or between, the great mass of muscles in the lower portions of the lumbar regions, for the purpose of making a counter incision into a small channel, the course of which we did not and could not know, even approximately, would have been, under any circumstances, an unjustifiable procedure—and especially so in the case of the President, whose hold upon life during all this long period seemed to depend upon a thread.

---

ON THE TREATMENT OF THE VARIOUS FORMS OF CONSUMPTION. BY ROBERTS BARTHOLOW, M.D., LL.D., Professor of Materia Medica and Therapeutics in the Jefferson Medical College, Philadelphia.

I will take it for granted, as is generally believed, that there are three distinct forms of pulmonary consumption: Chronic, catarrhal, or caseous pneumonia, which is an essentially inflammatory disease; chronic tuberculosis, with special direction to the pulmonary parenchyma, which is a diathetic malady, and fibroid phthisis, which is a chronic interstitial pneumonia ingrafted on a chronic bronchitis. It must be obvious that if this classification has a proper basis, it does not suffice to propose a plan of treatment for consumption, but our therapeutical methods must be guided and controlled by the pathological conditions. Whilst this is true, there are certain general principles of treatment applicable to all forms. I propose to consider these first—they are climate and personal hygiene.

In considering the subject of a suitable climate for a pulmonary invalid, I will not go beyond the limits of the United States, within which are contained the utmost variety, and, indeed, the perfection of health resorts for this purpose. In the absence of any statistical data showing the results of prolonged residence in particular localities which might indeed settle the question, we have some general principles to guide us, too little regarded by the profession, but of great value. We owe to Dr. Bowditch, of Boston, the eminent physician and sanitarian, the first principle, which he established for Massachusetts, and which has been confirmed on a larger scale for England. The Bowditch generalization is, that there is a constant ratio between the number of cases of consumption and the amount of water—rainfall, and collections of water in streams, ponds, and lakes. This principle is not applicable to the ocean, where other conditions obtain. Certain parts of England, having had a large mortality from consumption, present a very different report since suitable drainage works have been put into operation. You need only to cast your eye over the elaborate Atlas of Medical Geography, by Lombard, to see how large a part excess of moisture plays in the geographical distribution of phthisis. All along the sea-coast are traced the deeply shaded lines, whilst in the elevated interior regions, the mortality has disappeared. It is true that density of population and other evil hygienic influences are at work, but excess of moisture is a large factor.

Next to dryness of soil and climate as a remedy for consumption is elevation. The fact stands out as conspicuously in the great Atlas of Medical Geography as the previously considered influence. In the elevated regions—the great plains and plateaus of India, Africa, and America—phthisis is almost unknown (Lombard, vol. iv., p. 420.) Elevation has an important influence in the relief of phthisis, because the air is dry and rarefied. Breathing rarefied air lessens intrathoracic pressure, increases the rate of the respiratory movements and the rapidity of the pulmonary circulation. Residence in a rarefied atmosphere increases the rapidity of the circulation and the amount of blood in the peripheral vessels. The influences of these factors in promoting digestion, assimilation, and tissue metamorphosis are unquestionable. Uniformity is only less important as a requisite for a climate for pulmonary invalids. The reason of this requirement is obvious. Those invalids in a condition to be benefited by outdoor exercise need an equable temperature in which to pursue their sports or recreations with safety. But more important than this is the bad influence of a variable climate in causing attacks of bronchial catarrh, a morbid process so much concerned in the production of caseous phthisis. Applying these principles to the question of a climate for consumptives, I place first on the list the great plains and plateaus of our interior continent, next certain parts of California, then a limited district, of which Aiken, South Carolina, may be regarded as the center, and lastly, the upper lakes and Minnesota, and the Red River of the North. In the limited space of such a lecture it is quite impossible to go further into details on the subject of climate.

Amongst the questions of personal hygiene alimentation, is the most important. And here, gentlemen, I have some important facts to submit to your consideration. We owe to Hughes Bennett the important practical suggestion that the initial disorders of phthisis are stomàchal—that before any pulmonary trouble arises there is a period during which the organs of digestion do poor work, prepare the aliment imperfectly, and hence the nutrition of the body declines. That acute minded physiological surgeon, Mr. Jonathan Hutchinson, has investigated this, as so many other subjects, in a quite original manner, and he finds that almost universally a form of dyspepsia precedes phthisis. In fact it is a matter of common observation, that the individual is out of condition, is losing ground, and this long before the manifestations of pulmonary lesions of any kind. A chronic gastro-intestinal catarrh is present, and one of its special features is that the patient has a positive distaste for the fatty constituents of the food, and that the power to digest fats is much impaired. In a disease characterized by failure of nutrition, and in which the process of repair is so feeble, it is of the utmost importance to maintain the digestion and assimilation at the highest efficiency. There is a general agreement on this point, and hence no sooner is the diagnosis of phthisis arrived at than the injunction of active feeding follows. Without any attempt at correcting the morbid state of the digestive organs, they are plied with the richest hydrocarbons, with a teaspoonful of cod-liver oil before each meal, by way of appetizer, and a tablespoonful or more of whisky after meals as a digester. It results from this method that the appetite is utterly destroyed, and the food, swallowed with disgust, is either rejected or remains to distress. I hope you will always bear in mind that it is not the quantity of food swallowed, but the amount digested and assimilated which is important to the welfare of the individual. The diet, then, should be carefully regulated to the requirements of the individual. Let me now give you a practical hint, on which you will be able to act with great good to your patients. *A period of rest to the*

*stomach by a minimum diet will enable the organ to recover from the morbid state, and will induce a great appetite.* Acting on this rule, I have repeatedly effected great improvement in the condition of phthisical subjects at the initial period. Put the patient on milk for a week or two, and then gradually construct a suitable dietary. To remove the condition of gastro-intestinal catarrh there are several remedies on which we may rely with confidence. Arsenic stands first, probably, and is to be given in small doses—two drops—three times a day, before meals. Scarcely inferior to arsenic is the combination of iodine and carbolic acid: ℞. Tinct. iodinii. ʒj. acid. carbol. ʒss. M. Sig. One or two drops in a tablespoonful of water three times a day, before meals. The oxide and nitrate of silver are also highly serviceable, but unfortunately they cannot be continued long, owing to the danger of argyria. The mineral acids, if the digestion merely flags, or if the previous remedies have been taken for a sufficient time, are very important remedies. I have seen some striking results from the use of the nitro-muriatic acid during the initial stage, but the action of this remedy in changing morbid states of the mucous membrane and in promoting appetite and digestion is the secret of any curative power it possesses. The alkaloid strychnia, dissolved in diluted muriatic acid, makes a useful combination, for strychnia is probably the most effective agent which we possess for arresting the vomiting of phthisis.

If the morbid state of the mucous membrane has been removed, and the appetite restored, then we may consider the question of forcing the nutrition by increasing the performance of the assimilative organs. Now we may give cod-liver oil, and aid its digestion by the administration of malt liquors or alcohol in some form: It should be given after meals, to be digested and assimilated with the other foods. It is a great error to administer the oil, as is so often done, on an empty stomach and some time before the meal, because it will spoil the appetite, and keep the stomach at work during its proper period of repose. Furthermore, the quantity of oil taken should rarely exceed a teaspoonful, for this amount only can the digestive organ properly assimilate at one time. If it impair the appetite and cannot be digested, it is useless, and should not be continued. Bernard has put us in possession of an important fact, which ought always to be utilized—that is, that ether promotes the digestion of the oil when administered with it. Bernard's notion was that ether increases the flow of the pancreatic juice, and must therefore promote the emulsionizing of the oil. Whether or no the theory be correct, there has been accumulated sufficient testimony to show that the digestion of the oil is much more easily and perfectly accomplished by the addition of some minims of ether.

To what extent is the administration of alcohol desirable and proper in the treatment of phthisis? First of all, we should take our position firmly against a too prevalent notion that whisky is in a certain degree antidotal, and that any case of consumption must be cured if only it is given early enough, and in sufficient quantity. The mischievous fallacy is so far from true, that we now know a form of degeneration of the pulmonary parenchyma is produced by alchoholic excess. Before hectic comes on, as a rule, the extract of malt and malt liquors are preferable to whisky and brandy. When wasting proceeds rapidly, and destruction of the lung tissue is going on at the same pace, when the fever approaches more and more the septicæmic type, the stronger liquors are better, but at any period whisky should be given instead of malt liquors, if it agrees better. Is there any guide to the proper administration of alcohol? I am perfectly clear that these are well-defined principles. That quantity of spirit which increases the appetite, and improves the digestion, is the proper quantity. An excess is hurtful, because the alcohol precipitates the pepsin from its solution in the gastric juice, and therefore suspends the production of peptones. A large quantity taken on an empty stomach is without influence on the digestion of foods, and whilst it affects the mucous membrane injuriously, after absorption acts on the hepatic cells. These reasons seem conclusive in favor of a moderate quantity of alcoholic food—say half an ounce of whisky—taken after each meal.

[CONCLUDED IN JANUARY NUMBER.]

